# Multimodal Imaging of Isolated Foveal Hypoplasia: A Case Report

**DOI:** 10.4274/tjo.galenos.2020.58638

**Published:** 2020-10-30

**Authors:** Cumali Değirmenci, Filiz Afrashi, Serhad Nalçacı, Onur Furundaoturan

**Affiliations:** 1 Ege University Faculty of Medicine, Department of Ophthalmology, İzmir, Turkey

**Keywords:** Albinism, foveal hypoplasia, optical coherence tomography, optical coherence tomography angiography

## Abstract

Foveal hypoplasia is characterized by the lack of development of a normal fovea. It may be isolated or may occur secondary to ocular conditions. Optical coherence tomography (OCT), fluorescein angiography, fundus autofluorescence, and OCT angiography may be used for diagnosis. In this case report, we present a patient with foveal hypoplasia that was diagnosed with multimodal imaging.

## Introduction

Foveal hypoplasia is defined as the underdevelopment of the fovea and is characterized by nystagmus and low visual acuity. It is usually associated with optic nerve hypoplasia, familial exudative vitreoretinopathy (FEVR), Stickler syndrome, albinism, aniridia, and microphthalmia. It may also be isolated with no clear etiology. The visual acuity of patients with foveal hypoplasia varies from 20/20 to 20/50 and they have no foveal depression or pigmentation. Some authors have reported that foveal hypoplasia is related to mutations of genes such as P*AX6*, OCA2, and GPR143 that are associated with albinism.^1,2^

The normal fovea is observed to have a central depression with loss of the inner retinal layer and lengthening of the outer segments of the photoreceptors on optical coherence tomography (OCT). Additionally, OCT angiography (OCTA) revealed that the fovea has a central avascular black gap. A staging system based on OCT was developed for patients with foveal hypoplasia.^3,4^ The current study presents the multimodal imaging of a patient with foveal hypoplasia.

## Case Report

**Figure 1:**
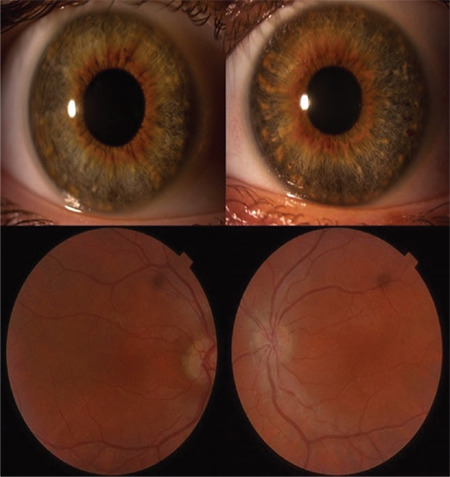
Normal anterior segment photos of the patient without any loss of pigmentation or iris transillumination (upper panels) and fundus images of the patient without foveal reflex (lower panels)

A 19-year-old man presented to the clinic with complaints of non-progressive low visual acuity. The patient had no previous major illnesses and did not report any similar family history. On ophthalmic examination, his best corrected visual acuity (BCVA) was 0.40 logMAR with +5.50-1.50x135 in the right eye and 0.10 logMAR with +5.50-2.00x110 in the left eye. There was no manifest or latent strabismus, but latent nystagmus was found in both eyes. Examination of the anterior segment did not show any indicators of ocular albinism such as transillumination, and iris pigmentation was normal. Funduscopic examination revealed a normal optic disc and vessels but a lack of foveal pigmentation (Figure 1).

**Figure 2:**
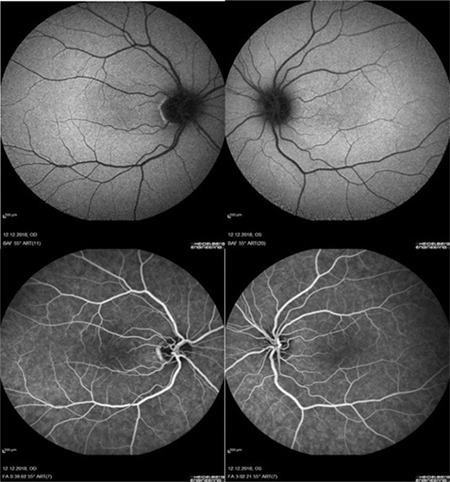
Fundus autofluorescence demonstrated a lack of foveal hypoautofluorescence in the central macula (upper panels) and fundus fluorescein angiography images did not show foveal avascular zone (lower panels)

**Figure 3:**
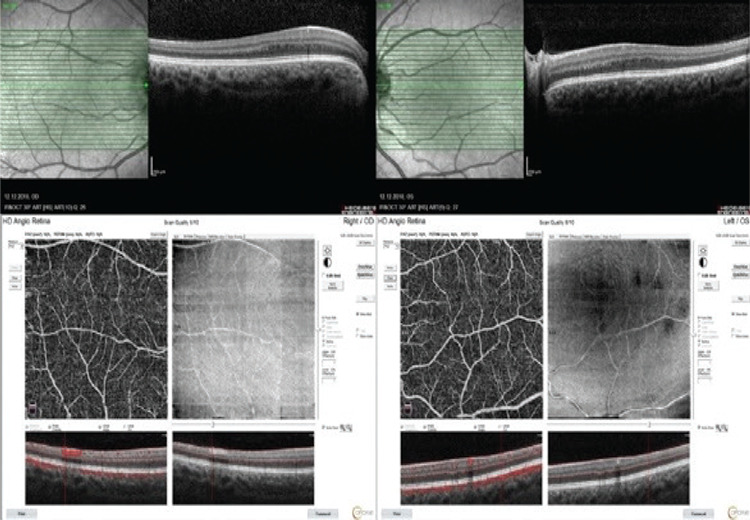
Optical coherence tomography showed no foveal depression (upper panels) and optical coherence tomography angiography images revealed the lack of foveal avascular zone (lower panels)

OCT images showed no foveal depression in either eye with continuity of the inner retinal layers. The outer segments of the photoreceptors were not lengthened. Fluorescein angiography showed the foveal avascular zone was absent. OCTA confirmed the lack of a foveal avascular zone along with absence of the central black gap. Fundus autofluorescence images also showed no hypoautofluorescence in the corresponding fovea (Figure 2, 3).

The patient was informed about this publication and written consent was obtained to publish collected images.

## Discussion

In the current report, we present a patient with isolated foveal hypoplasia associated with an underdeveloped fovea and nystagmus without any etiology such as optic nerve hypoplasia, FEVR, Stickler syndrome, albinism, aniridia, microphthalmia, nanophthalmus, retinopathy of prematurity, incontinentia pigmenti, or achromatopsia.

Patients with foveal hypoplasia present with decreased visual acuity. Thomas et al.^2^ proposed a grading system for foveal hypoplasia to predict the prognosis of visual acuity based on OCT scans. The authors suggested 3 key points for the arrest of foveal development, including incursion of the plexiform layer to the posterior of the foveola, partial displacement of the inner retinal layers, and lengthening of the outer segment of photoreceptors. In the current case, the patient had no foveal pit or lengthening of the outer segment of the photoreceptors; both of which correspond to the worst prognosis. Also, outer nuclear layer widening was not observed. Based on these findings, the patient had grade 4 foveal hypoplasia. However, the BCVA of the patient was 0.4 logMAR in the right eye and 0.1 logMAR in the left eye. It is possible that structural properties of the fovea and visual acuity may not be correlated; further investigations are needed to better understand this.^3^

Fluorescein angiography or fundus autofluorescence are techniques used to establish the lack of the foveal avascular zone and foveal hypoautofluorescence. OCTA is an easy, rapid, and noninvasive method for imaging the retinal microvasculature and can reveal the lack of foveal avascular zone in the superficial and deep capillary layers in foveal hypoplasia.^5,6,7^ In the current case, the patient showed no foveal avascular zone in both eyes.

Although foveal hypoplasia can be determined and graded by OCT, different imaging techniques including fundus autofluorescence, fluorescein angiography, and OCTA can be used to perform further evaluation. It is recommended that patients with low visual acuity are evaluated with multimodal imaging methods.
